# Sustained suppression of viral replication in improving vitamin D serum concentrations in patients with chronic hepatitis B

**DOI:** 10.1038/srep15441

**Published:** 2015-10-21

**Authors:** En-Qiang Chen, Lang Bai, Tao-You Zhou, Min Fe, Dong-Mei Zhang, Hong Tang

**Affiliations:** 1 Center of Infectious Diseases, West China Hospital, Sichuan University, Chengdu, Sichuan 610041, PR China; 2 Division of Infectious Diseases, State Key Laboratory of Biotherapy, Sichuan University, Chengdu, Sichuan 610041, PR China

## Abstract

Recently, the role of vitamin D in chronic hepatitis B (CHB) has attracted a lot attention. In this study, 128 naïve CHB patients (91 with positive HBeAg, 37 with negative-HBeAg) were enrolled, and 128 volunteers without liver diseases were enrolled as controls. Compared to that of healthy controls, the mean level of 25(OH)D_3_ in CHB patients was significantly lower; and the percent of patients with sufficient 25(OH)D_3_ (≥20 ng/mL) was also significantly lower than that of healthy controls. Among those CHB patients, the level of 25(OH)D_3_ was negatively correlated with the serum HBV-DNA level. Additionally, the level of 25(OH)D_3_ was significantly lower in HBeAg-positive patients than that in HBeAg-negative patients. After the patients went through the long-term antiviral treatments, both the mean level of 25(OH)D_3_ and the percent of patients with sufficient 25(OH)D_3_ increased significantly. Additionally, patients who were HBeAg free after the treatment also had much higher 25(OH)D_3_ level than those with persistent positive HBeAg. All those data suggested that the low vitamin D serum level was dangerous for CHB patients, and the level of 25(OH)D_3_ was highly negatively correlated with HBV-DNA levels. Effective antiviral therapy might increase the level of vitamin D in CHB patients.

Though the role of vitamin D in calcium regulation and bone homeostasis has been well recognized^1^, the role of vitamin D on modulating the innate and adaptive immune responses attracted great interests of researchers^2^ recently, especially after the discovery of its ligand-vitamin D receptors on immune cells (such as B cells, T cells, and antigen-presenting cells). It is now clear that vitamin D deficiency is associated with increased autoimmunity and susceptibility to infections^3^.

Recently, vitamin D was also reported to play an emerging role in viral hepatitis^4^,^5^. For example, chronic hepaitis C (CHC) patients frequently suffer from severe vitamin D deficiency^6^,^7^; and this deficiency may be associated with liver fibrosis and low rate of sustained virologic response to PegIFNα-based therapy^8^. Interesting, Farnik and his colleagues recently reported that low concentration of vitamin D was also associated with high levels of HBV replication in CHB patients^9^. However, this correlation between vitamin D concentration and HBV DNA replication was not observed in a later study^10^. In addition, it remains unclear whether there is a relationship between vitamin D status and antiviral response of CHB patients. Thus, more researches were needed to investigate the role of vitamin D in CHB patients.

Cholecalciferol is the precursor of the bioactive vitamin D metabolite calcitriol (also called 1,25(OH)_2_D_3_). Cholecalciferol is hydroxylated to 25(OH)D_3_ at position 25 in the liver followed by hydroxylation at position 1 in the kidney to become bioactivated calcitriol. Unfortunately, therer are no reliable clinical assays to quantify calcitriol^11^. Therefore, the stable and easy-to-quantify metabolite 25(OH)D_3_ is usually measured clinically to evaluate a patient’s vitamin D status. To develop a deeper understanding of vitamin D status in CHB, this study was designed to observe the distribution of serum v25(OH)D_3_ in CHB cohorts, investigate the correlation of serum vitamin D distribution with HBV-related parameters, and reveal whether an effective long-term antiviral therapy would help to improve the vitamin D deficiency or insufficiency.

## Results

### General characteristics

The baseline demographic characteristics and laboratory evaluations of the CHB patients and healthy controls were summarized in Table 1. The distributions of age, gender and BMI were comparable between CHB patients and healthy controls. The 128 naïve CHB patients were relatively young with the mean age at 33.57 ± 8.47 years, and there were more male patients than female patients (71.88% *vs*. 28.12%). 91 patients (71.09%) were HBeAg positive; and HBV genotype B (64.84%) was predominant.

### The distribution pattern of serum 25(OH)D levels in CHB patients

**Figure 1:**
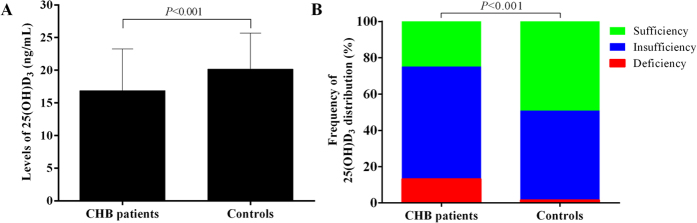
The distribution pattern of serum 25(OH)D_3_ in CHB patients and healthy controls. (**A**) the mean level of serum 25(OH)D_3_; (**B**) the distribution interval of serum 25(OH)D_3_.

As shown in Fig. 1, the mean level of 25(OH)D_3_ in CHB patients was 16.88 ± 6.40 ng/mL. 13.28% (17/128) patients had vitamin D deficiency, and 61.72% (79/128) patients had vitamin D insufficiency, while only 25.00% (32/128) had adequate vitamin D. On the other hand, the mean level of 25(OH)D_3_ in healthy controls was 20.16 ± 5.50 ng/mL. 1.56% (2/128) of those people in the control group had vitamin D deficiency, and 49.22% (63/128) had vitamin D insufficiency, while 49.22% (63/128) patients had adequate vitamin D. As shown above, the mean serum level of 25(OH)D_3_ in CHB patients was significantly lower than that for healthy controls (*P* < 0.001). The percentage of CHB patients with sufficient 25(OH)D_3_ was also significantly lower than that in healthy controls (*P* < 0.001). All those results showed that the reduced vitamin D serum level was more common in CHB patients than that in general populations without CHB.

### Relationship between serum 25(OH)D_3_ levels and clinical parameters

**Figure 2:**
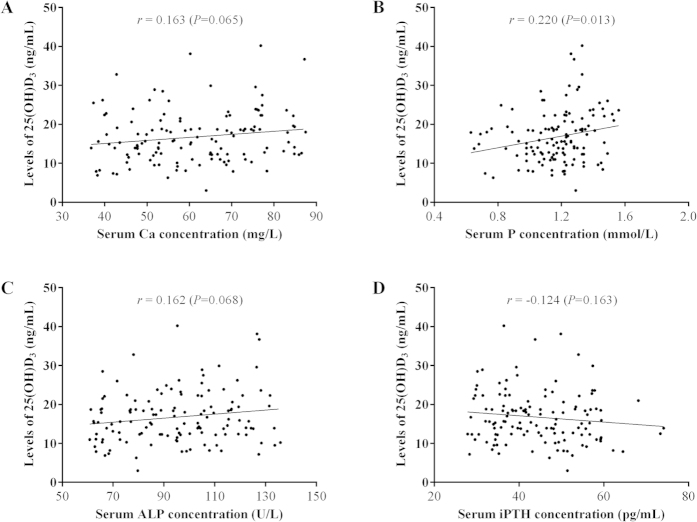
The distribution and correlation of serum 25(OH)D_3_ with serum Ca (**A**), P (**B**), ALP (**C**) and iPTH (**D**) .

**Figure 3:**
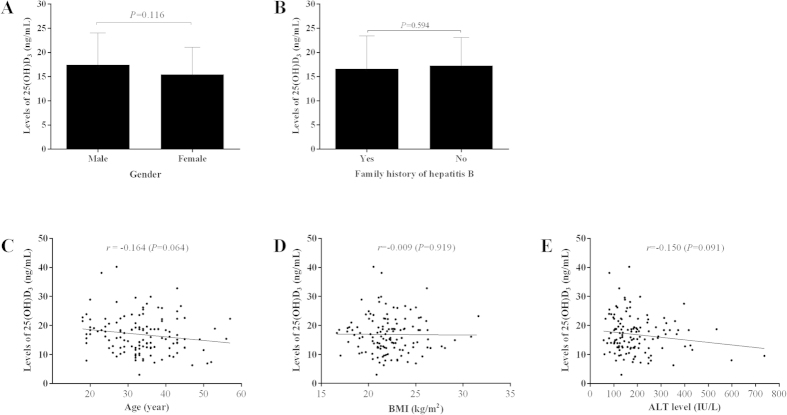
The distribution and correlation of serum 25(OH)D_3_ levels with demographic and biochemical parameters. (**A**) the distribution of serum 25(OH)D_3_ based on gender (**A**) or family history of hepatitis B (B); the correlation of serum 25(OH)D_3_ level with patient age (**C**), BMI (**D**), and serum ALT levels (**E**).

**Figure 4:**
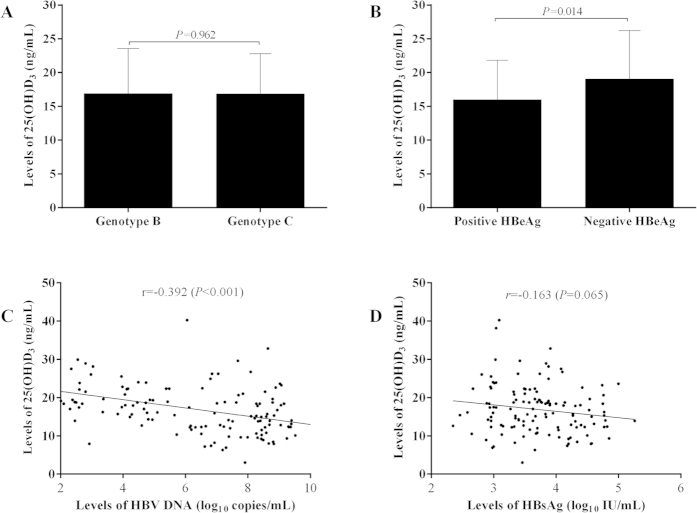
The distribution and correlation of serum 25(OH)D_3_ levels with virological parameters. (**A**) the distribution of serum 25(OH)D_3_ among different HBV genotypes (**A**) or HBeAg states (**B**); the correlation of serum 25(OH)D_3_ levels with patient serum HBV-DNA (**C**) and HBsAg levels (**D**).

As shown in Fig. 2, the serum 25(OH)D_3_ level was not significantly correlated with either serum Ca (*r* = 0.163, *P* = 0.065), P (*r* = 0.220, *P* = 0.013), ALP (*r* = 0.162, *P* = 0.068), or iPTH level *(r* = −0.124, *P* = 0.163). The distribution pattern of serum 25(OH)D_3_ level in CHB patients was not significantly correlated with patient age (*r* = −0.164, *P* = 0.064), gender (*P* = 0.116), BMI (*r* = −0.009, *P* = 0.919), family history of hepatitis B (*P* = 0.594), or serum ALT levels (*r* = −0.150, *P* = 0.091) either (Fig. 3). For HBV-associated variables shown in Fig. 4, there was no significant difference in serum 25(OH)D_3_ between genotypes B and C (16.90 ± 6.66 *vs*.16.84 ± 5.97 ng/mL, *P* = 0.962), and no significant correlation between 25(OH)D_3_ and quantitative HBsAg titer (*r* = −0.163, *P* = 0.065). However, the serum 25(OH)D_3_ level was significantly negatively correlated with the serum HBV-DNA level (*r* = −0.392, *P* < 0.001), and the serum 25(OH)D_3_ level was also significantly lower in HBeAg-positive patients than in HBeAg-negative patients (15.99 ± 5.86 *vs*. 19.05 ± 7.20 ng/mL, *P* = 0.014). Those results indicated that low 25(OH)D_3_ in CHB patients might be caused by the high viral replication.

### The change of 25(OH)D_3_ level after antiviral treatment

**Figure 5:**
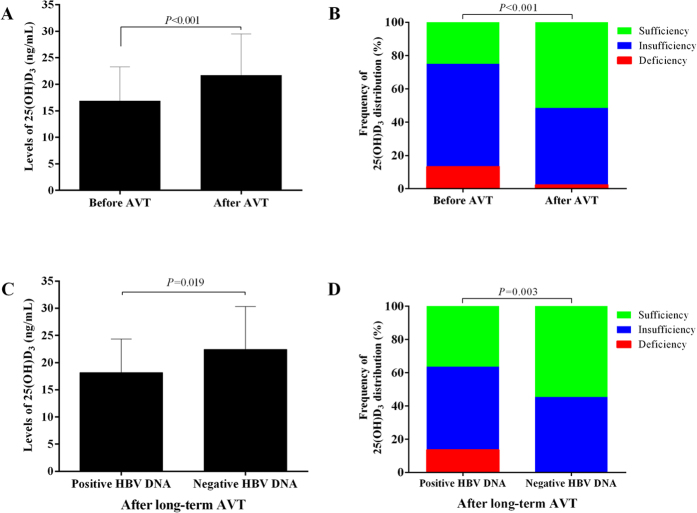
serum 25(OH)D_3_ in patients before and after the long-term antiviral treatment. (**A**) the mean level of serum 25(OH)D_3_ before and after treatment; (**B**) the distribution interval of serum 25(OH)D_3_ before and after treatment; (**C**) the mean level of serum 25(OH)D_3_ between patients with detectable and undetectable HBV DNA after treatment; (**D**) the distribution interval of serum 25(OH)D_3_ between patients with detectable and undetectable HBV DNA after treatment.

All CHB patients were treated with oral nucleoside/nucleotide analogues, and the duration of antiviral therapy ranged from 5 to 6 years. After the antiviral treatment, serum 25(OH)D_3_ in CHB patients increased significantly with the mean level raising from 16.88 ± 6.40 ng/mL to 20.16 ± 5.50 ng/mL (Fig. 5A, *P* < 0.001). After the treatment, only 2.34% (3/128) patients still had vitamin D deficiency, while 46.09% (59/128) patients became vitamin D insufficiency, and 51.56% (66/128) patients already had adequate vitamin D (Fig. 5B).

**Figure 6:**
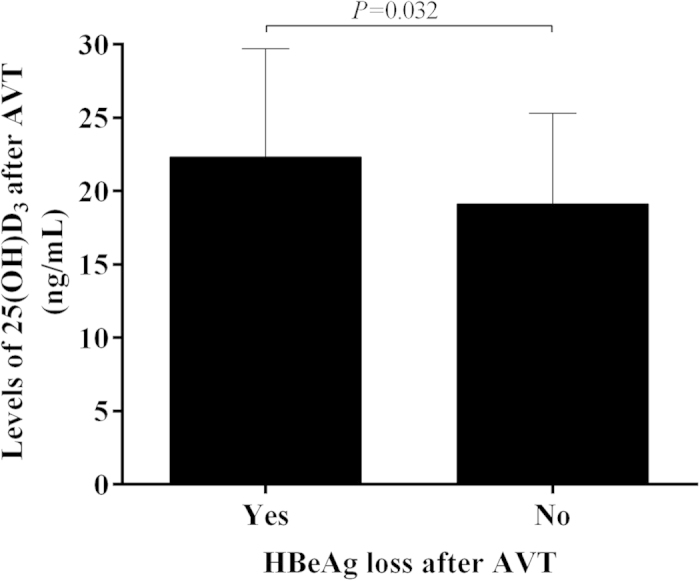
The mean level of serum 25(OH)D_3_ between patients with HBeAg free or not after treatment.

After the 5 ~ 6 year antiviral treatment, 82.81% (106/128) patients achieved undetectable HBV DNA, while 17.19% (22/128) patients still had detectable HBV DNA. Both mean 25(OH)D_3_ level (22.42 ± 7.94 *vs*. 18.16 ± 6.20 ng/mL, *P* = 0.019) and percent of patients with sufficient 25(OH)D_3_ [54.72(58/106) *vs*. 36.36%(8/22), *P* = 0.003] were significantly higher in patients with undetectable HBV DNA (Fig. 5C,D) than in patients with detectable HBV DNA. In addition, among patients with initial positive-HBeAg, those who achieved HBeAg free during the antiviral treatment also had much higher 25(OH)D_3_ than those with persistent positive HBeAg (22.33 ± 7.39 *vs*. 19.12 ± 6.19 ng/mL, *P* = 0.032)(Fig. 6). Those results suggested that sustained and effective suppression of HBV DNA replication would help to increase the serum 25(OH)D_3_ level.

### The seasonality of serum 25(OH)D_3_ levels

**Figure 7:**
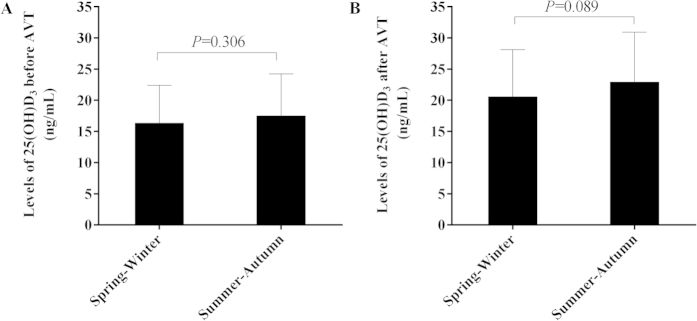
The relationship of serum 25(OH)D_3_ levels with seasonal changes before and after treatment.

Previous study showed that serum 25(OH)D_3_ levels fluctuated seasonally due to the change of sunlight exposure for Caucasian. To determin whether this phenomenon also existed in our study, we further investigated the seasonality of serum 25(OH)D_3_ levels for patients in our study (Spring-Winter *vs*. Summer-Autumn). As shown in Fig. 7, there was no significant difference in serum 25(OH)D_3_ level between Summer-Autumn (n = 61) and Spring-Winter (n = 67), for patients either before (17.49 ± 6.73 *vs*. 16.32 ± 6.08 ng/mL, *P* = 0.306) or after (22.92 ± 7.99 *vs*. 20.56 ± 7.54 ng/mL, *P* = 0.089) the antiviral treatment. Our result showed that seasonal changes had limited influence on serum 25(OH)D_3_ levels in the population with balanced sunlight exposure throughout the year.

## Discussion

In this study, the distribution pattern of serum 25(OH)D_3_ and the relationship of serum 25(OH)D_3_ with demographic and laboratory parameters were analyzed in NAs-treated naïve CHB patients. The key findings from our study are: (1) a significantly higher portion of naïve CHB patients had vitamin D deficiency and insufficiency than the healthy controls, (2) a significantly negative correlation of serum 25(OH)D_3_ and HBV-DNA levels, (3) a lower level of vitamin D in HBeAg-postive patients than in HBeAg-negative patients before the antiviral treatment, (4) sustained and effective suppression of HBV DNA replication would help to increase the serum 25(OH)D_3_ level.

Recent studies have shown that the low vitamin D level is common in CHB patients. For example, Dr. Farnik H *et al*. reported that there were up to 34% patients with severe vitamin D deficiency and 47% patients with vitamin D insufficiency^9^. However, it is unclear whether the low vitamin D is more common in CHB patients than in general population, because there is no clinical trial data on this topic. Our study was the first one to compare vitamin D levels between CHB patients and healthy controls. Though the sample size was relative small, the findings were significant. Our research showed that vitamin D levels were much lower in naïve CHB patients than in general populations, which suggested HBV persistent infection might exacerbate the vitamin D decline. And it was reported that immune-mediated suppression of liver 25-hydroxylases might lead to vitamin D deficiency in patients with viral hepatitis^6^,^12^. However, the exact mechanism still requires further investigation.

In our study, 75% CHB patients from West China (Chengdu City) had either vitamin D deficiency or vitamin D insufficiency. The proportion of patients with low vitamin D was significant higher than that in another similar study performed in South China (only 8.7% of CHB patients with 25(OH)D_3_ <20 ng/mL)^10^. Because Chengdu is a basin surrounded by mountains in West China, patients had shorter sunshine time than those in other parts of China. Therefore, besides the chronic HBV infection, the inadequate ultraviolet light exposure should also be an important risk factor for lower vitamin D level in this cohort. It was also reported that there was a seasonal fluctuation of serum vitamin D^9^. However, the seasonal fluctuation of serum 25(OH)D_3_ (Spring-Winter *vs*. Summer-Autumn) was not observed in our study either before or after the patients went through the long-term antiviral treatment. As far as we know, majority of CHB patients in this study were perennially engaged in indoor works with very limited outdoor sunshine exposure. The weak seasonality might be due to the little sunlight exposure.

Recently, Prof. Hou JL *et al*. reported that serum 25(OH)D_3_ was not associated with viral levels in patients with high HBV-DNA^10^. However, in our study, there was a significantly negative correlation of 25(OH)D_3_ with HBV-DNA levels. And our finding was also supported by the finding from Prof. Farnik and his colleagues^9^. In our study, patients had different levels of HBV DNA ranging from 1.47 to 10.09 log_10_ IU/mL. In the cohort reported by Prof. Hou JL, the mean level of HBV DNA was relatively higher (7.22 ± 1.48), with majority above 4 log_10_ IU/mL. Therefore, we believe that the inconsistent correlation between vitamin D and HBV DNA in different studies should be explained by different distribution patterns of HBV DNA levels.

Interestingly, we have found that early HBeAg-positive patients had lower serum 25(OH)D_3_ than HBeAg-negative patients did. Those patients who achieved HBeAg free after the antiviral treatment also had significantly higher 25(OH)D_3_ than those with persistent positive HBeAg. HBV can manipulate and modulate the immune response to achieve persistent infection, and HBeAg is one of key viral proteins involved in these processes^13^,^14^. Liver is an important and indispensable organ during the metabolic processes of vitamin D and the life-cycle of HBV. It’s possible that the persistent existence of high titer HBeAg may indirectly lower the production of active metabolite of vitamin D.

It is worth to mention that high necroinflammatory activity might also be responsible for the low vitamin D level in CHB patients^15^ and that the necroinflammatory activity was loosely related to the HBV replication and HBV-mediated immune responses. In our study, patients who achieved sustained viral response seemed to have a significantly higher mean vitamin D level, with higher percent of patients with sufficient 25(OH)D_3_. In addition, the improvement of vitamin D level was more obvious in patients who were HBeAg free. Therefore, we believe that an effective antiviral therapy may help to improve the vitamin D deficiency or insufficiency.

In conclusion, we reported here that naïve CHB patients had significantly lower 25(OH)D_3_ and that the reduced 25(OH)D_3_ could be improved after the patients went though a long-term effective antiviral therapy. That being said, further large scale, multi-center trials are still needed to confirm and expand these preliminary findings.

## Methods

### Patients

128 CHB patients (treatment-naïve patients with 91 positive HBeAg patients, 37 negative-HBeAg patients) were recruited from the outpatient department of West China Hospital, and all of them met the general indications for antiviral treatment. Serum samples were collected at the patients’ first visit at our outpatient clinic between June 2007 and January 2009 as well as at their follow-up visits during the five or six-year antiviral treatment during follow up. All samples were stored at −70 °C. Patients were excluded if they had co-infections (HCV and HIV), other concomitant liver diseases such as autoimmune liver disease, decompensated liver cirrhosis, hepatocellular carcinoma, liver transplant, or immunosuppressive medication. A total of 128 healthy volunteers were also enrolled as controls.

This research was approved by Ethics Committee of West China Hospital of Sichuan University, and informed consent was obtained from each patient or healthy volunteer. Present study was also in compliance with the ethical guidelines of the 1975 Declaration of Helsinki.

### Laboratory variables detection

25(OH)D_3_ levels in serum samples were measured using an automated electrochemiluminescence-based assay, Elecsys Vitamin D Total (Roche Diagnostics, Mannheim, Germany). The data were expressed in nanograms per milliliter. Serum 25(OH)D_3_ concentrations of <10 ng/mL, <20 ng/mL and ≥20 ng/mL were defined as vitamin D deficiency, insufficiency and sufficiency, respectively.

The serum calcium was measured with the inductively coupled plasma mass spectrometry, while the serum phosphorus concentration was measured by phosphomolybdic acid colorimetry method. Serum alanine aminotransferase (ALT) and alkaline phosphatase (ALP) were measured using the Automatic Biochemistry analyzer (Olympus AU5400, Olympus Corporation, Tokyo, Japan). Serum intact parathyroid hormone (iPTH) was measured by a two-site immunoradiometric assay. Serum HBV DNA was measured using the Cobas Taqman assay (Roche Diagnostics, Branchburg, NJ) with a lower limit of detection of 291 copies/mL. HBV genotype was measured by directly gene sequencing of HBV DNA S gene. Serum HBsAg titres were quantified according to research protocol using Elecsys® HBsAg II Quant Assay (Roche Diagnostics, Penzberg, Germany). Serum HBeAg was measured using commercially available immunoassays (Roche Diagnostics, Indianapolis, IN, USA).

### Statistical analysis

Statistical analysis of the data was performed using the statistical package SPSS (version17.0; SPSS, Inc., Chicago, IL). Continuous variables were presented as the mean ± SD and categorical variables were presented as frequencies (%). Levels of HBV DNA and HBsAg were transferred to log10IU/mL. Categorical variables were analyzed using χ2 test, or Fisher’s exact test when appropriate. Continuous variables with normal or skewed distribution were analyzed using Student’s or Mann-Whitney test, and the comparison of serum 25(OH)D_3_ level before and after antiviral treatment was accomplished using paired samples t test, with *P* value below 0.05 considered statistically significant. The correlation between two continuous variables was analyzed using Spearman’s bivariate correlation, and the correlation was significant with the level at 0.01 (2-tailed). All statistical analyses were done with SPSS Version 18.0 (SPSS, Chicago, IL), and all figures were drawn using GraphPad Prism 6 (GraphPad Software Inc., California, USA).

## Additional Information

**How to cite this article**: Chen, E.-Q. *et al*. Sustained suppression of viral replication in improving vitamin D serum concentrations in patients with chronic hepatits B. *Sci. Rep*. **5**, 15441; doi: 10.1038/srep15441 (2015).

## Figures and Tables

**Table 1:** Baseline Characteristics of CHB patients and healthy individuals.

	CHB patients (n = 128)	Controls (n = 128)	P-value
Age			
* Mean±SD (years)*	33.57 ± 8.47	35.17 ± 8.02	0.121
Gender			
* Male gender (n, %)*	92(71.88)	95(74.22)	0.673
Body mass index			
* Mean±SD (kg/m*^*2*^)	22.18 ± 2.63	22.75 ± 2.84	0.097
Family history of CHB			
* Positive (n, %)*	74(57.81)	0(0.00)	0.000
Serum ALT			
* Mean±SD (×ULN, IU/mL)*	3.83 ± 2.18	0.58 ± 0.30	0.000
HBV genotype			
* B/C (n, %)*	83(64.84)/45(35.16)	/	
Serum HBV DNA			
* Mean±SD (log*_*10*_ *IU/mL)*	6.38 ± 2.32	/	
Quantitative HBsAg			
* Mean±SD (log*_*10*_ *IU/mL)*	3.71 ± 0.62	/	
HBeAg statue			
* Positive, n(%)*	91(71.09)	/	
